# Two novel trimethoprim resistance genes, *dfra50* and *dfra51*, identified in phage-plasmids

**DOI:** 10.1128/aac.01695-24

**Published:** 2025-06-12

**Authors:** Kai Wang, Jikai Xu, Xiaowei Lu, Pan Yin, Li Chen, Ziwei Zhao, Alejandra Bravo, Mario Soberón, Jinshui Zheng, Ming Sun, Donghai Peng

**Affiliations:** 1 National Key Laboratory of Agricultural Microbiology, Huazhong Agricultural University 124443, Wuhan, Hubei, China; 2 College of Life Science and Technology, Huazhong Agricultural University 47895, Wuhan, Hubei, China; 3 Instituto de Biotecnología, Universidad Nacional Autónoma de México 7180, Mexico City, Mexico; 4 College of Informatics, Huazhong Agricultural University 627750, Wuhan, Hubei, China; Johns Hopkins University School of Medicine, Baltimore, Maryland, USA

**Keywords:** antibiotic resistance genes, phage-plasmids, dfrA, trimethoprim resistance

## Abstract

Phage-plasmids carry a significant burden of clinically relevant antibiotic resistance genes (ARGs). Intriguingly, the majority of these ARGs are found within plasmids with phage features, with a single exception residing in a phage genome with plasmid features. Therefore, we speculate that phage genomes with plasmid features, whose sequences are highly homologous to bacterial plasmids, may carry novel ARGs. We subsequently identified 46 such phage genomes by employing Hidden Markov models (HMMs) based on plasmid-specific protein profiles andbasic local alignment search tool (BLASTn) searches against the National Center for Biotechnology Information (NCBI) RefSeq Plasmid Database. Among them, six phages harbored seven ARGs identified through a lenient-threshold search strategy, of which only two had been previously reported. The remaining five ARGs were categorized as novel ARGs since their encoded proteins differed from known ARGs. Notably, half of the phages carried trimethoprim-resistant *dfrA*-like genes. Functional studies characterized these genes and demonstrated that the expression of two of these *dfrA* genes (*dfrA50* and *dfrA51*) can confer resistance to trimethoprim in *Escherichia coli*. Through genome analysis, we found that these phages with plasmid features likely contributed to the natural dissemination of these *dfrA* genes, as evidenced by their widespread presence in plasmids across various pathogenic bacteria. These findings underscore the importance of identifying and monitoring ARGs encoded by phage genomes with plasmid features that also function as plasmids in bacteria, aiming to proactively address the antibiotic resistance challenges posed by these phage-mediated dissemination events.

## INTRODUCTION

Bacterial antimicrobial resistance (AMR) has emerged as a pressing and escalating epidemic, compromising the efficacy of microbial infection treatments, and posing a major global public health threat (1). An estimated 4.95 million deaths worldwide in 2019 were attributed to bacterial AMR, with 1.27 million directly resulting from infections caused by bacterial AMR (2). The predominant reason for the emergence of AMR bacteria is the acquisition of antibiotic resistance genes (ARGs) through horizontal gene transfer (HGT) (1).

Bacterial plasmids serve as the primary reservoir of ARGs (3), and in nature, they show widespread dissemination through conjugative transfer (1). Simultaneously, a growing body of research has identified ARGs within prophages or temperate phages (4, 5), demonstrating that these phages can facilitate the extensive dissemination of these ARGs in nature (4, 6). Additionally, a prevalent and abundant mobile genetic element, termed phage-plasmids, is hybrid genetic element possessing dual functionality as both plasmids and phages. This includes phages defined in GenBank entries with plasmid features and plasmids defined in GenBank entries with phage features (7). Phages with plasmid features are phages that carry genetic elements associated with plasmid replication and segregation. Similarly, plasmids with phage features are plasmids that contain genetic elements usually associated with phages (7). These phage-plasmids can replicate both as typical phages and in the form of plasmids (7). Recent research indicated that 4.2% of phage-plasmids harbored clinically relevant ARGs, promoting their widespread dissemination through lysogenic conversion (8, 9). In-depth analysis revealed that all identified ARGs were situated in plasmids with phage features, with the sole exception of *bla*_SHV-2_, which is encoded by *Escherichia* phage RCS47 with plasmid features (8). The number of ARGs carried by phage genomes with plasmid features may be underestimated, as phage-plasmids could potentially exist simultaneously in both phage and plasmid forms, possibly harboring previously undiscovered novel ARGs. Therefore, it is essential to reassess the diversity and distribution of ARGs in phages that contain plasmid features and act as plasmids in bacteria to strengthen the monitoring of these ARGs.

Trimethoprim, a synthetic antibiotic recommended by the World Health Organization (WHO), ranks as the fourth most commonly prescribed antibiotic for infections caused by pathogenic bacteria, such as *Escherichia coli*, *Salmonella* spp., and *Klebsiella* spp. (10, 11). Trimethoprim functions by inhibiting the activity of dihydrofolate reductase, encoded by the *folA* gene in bacteria (12). This inhibition suppresses the conversion of dihydrofolate to tetrahydrofolate, consequently disrupting the synthesis of purines and pyrimidines, ultimately impeding DNA synthesis and hindering microbial proliferation (10). Trimethoprim is widely employed for the treatment of urinary tract infections caused by several gram-positive and gram-negative pathogens and is listed in the World Health Organization’s updated essential medicines (10). Acquisition of *dfrA* genes is a common mechanism for bacteria to develop resistance to trimethoprim (13).

In this study, we identified 46 phage genomes with plasmid features, each displaying high sequence homology to bacterial plasmids, from a total of 12,443 phage genomes in the NCBI database. Using a lenient-threshold search strategy, we identified seven potential ARGs encoded by six phage genomes. Among them, only two ARGs (*bla*_TEM-1_ and *bla*_CTX-M-27_) were previously reported, whereas the remaining five exhibited ≥2% difference in amino acid sequence from known ARGs, classifying them as potentially novel ARGs (14). Given that half of the phages carried a *dfrA*-like gene that might confer trimethoprim resistance, we studied the functions of two genes (*dfrA50* and *dfrA51*) and found that their expression in *E. coli* conferred resistance to trimethoprim. It is concerning that these phages with plasmid features have spread their *dfrA* genes among diverse pathogenic bacteria. Our study unveiled novel functional ARGs in phage genomes with plasmid features, highlighting the need for vigilant monitoring of ARGs within phage-plasmids to mitigate potential resistance dissemination.

## RESULTS AND DISCUSSION

### Phages exhibiting plasmid features carry novel ARGs

**Fig 1:**
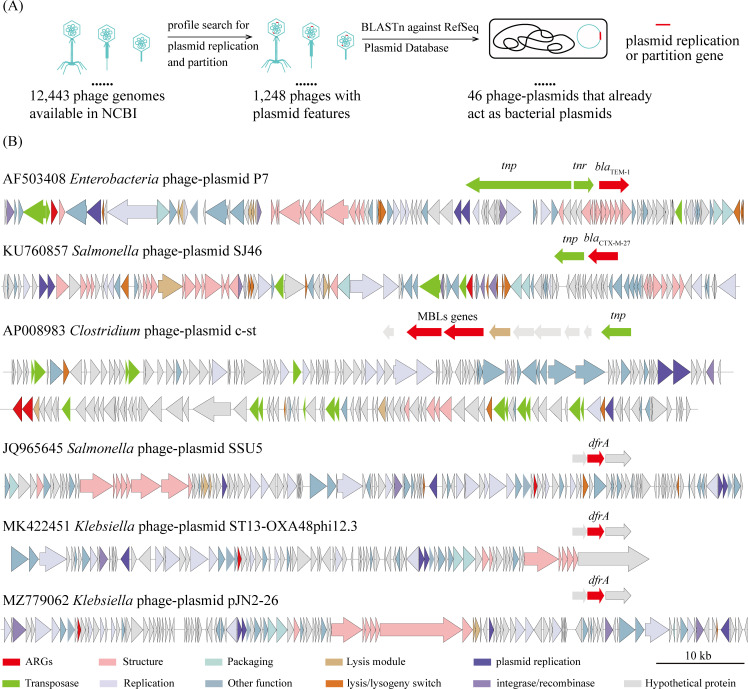
Identification of phage-plasmids that exist as plasmid forms in bacteria and the profile of the ARGs present in these phage-plasmids. (**A**) Procedure to identify phages with plasmid features (phage-plasmids) that also act as plasmids in bacteria. All available phage genomes were collected from the NCBI database. Protein profile HMMs specific for plasmid replication and partition systems were utilized to identify phages exhibiting plasmid features. BLASTn was employed to identify phage genomes whose sequences show high homology to bacterial plasmids. (**B**) Overview of the six identified phage-plasmids carrying ARGs and analysis of whether these ARGs are adjacent to mobile elements in each phage-plasmid. Gene functions are visualized in different colors, as indicated in the figure, with red representing ARGs. The *tnp* gene encodes transposase, *tnr* encodes Tn3 resolvase, *bla*_TEM-1_ and *bla*_CTX-M-27_ encode class A beta-lactamase, the MBL gene encodes metallo-β-lactamase, and the *dfrA* encodes dihydrofolate reductase (DHFR).

We gathered the sequences of 12,443 available phage genomes from the NCBI database to assess the diversity and distribution of ARGs in phage genomes containing plasmid features, whose sequences are highly homologous to bacterial plasmids. Utilizing the detection method outlined by Pfeifer et al. (7), we identified 1,248 phages with plasmid-related features, categorizing them as phage-plasmids. Subsequently, we employed BLASTn to align these phage-plasmid genomes against the nonredundant NCBI RefSeq Plasmid Database, revealing 46 instances of phage-plasmids capable of functioning as both phages and bacterial plasmids in natural environments (Fig. 1A).

Subsequently, we employed lenient threshold search strategies to unveil ARGs within the aforementioned 46 phage-plasmid genomes, revealing seven ARGs across six phage-plasmid genomes, including two reported genes encoding Serine-β-Lactamases (*bla*_TEM-1_ and *bla*_CTX-M-27_), two genes encoding Metallo-β-Lactamases (MBLs), and three *dfrA*-like genes encoding dihydrofolate reductases (DHFR) (Fig. 1B; Table S1). Notably, these genes exhibited ≥2% difference in their DNA or protein sequences with other reported ARGs, categorizing them as potential novel ARGs (14), except for the two, *bla*_TEM-1_ and *bla*_CTX-M-27_, which are located adjacent to genes encoding transposases (Fig. 1B).

### Expression of novel *dfrA* genes encoded by phage-plasmids confers trimethoprim resistance to *E. coli*

Interestingly, in addition to the two reported ARGs, we identified three novel *dfrA* genes in Enterobacterales. We investigated whether these phage-plasmid-carried *dfrA* genes could confer trimethoprim resistance to *E. coli*. Since QBP27508.1 from *Klebsiella* phage-plasmid ST13-OXA48phi12.3 differs from UAV85970.1 from *Klebsiella* phage-plasmid pJN2-26 by only four amino acids and shows 98.53% nucleotide identity (Fig. S1), we focused on QBP27508.1 for functional studies. The genes encoding AFL47010.1 from *Salmonella* phage-plasmid SSU5, QBP27508.1, and DfrA49 were synthesized and subcloned into pET21b(+) and pACYC184 vectors, respectively. These constructs were then used to evaluate their role in conferring trimethoprim resistance in *E. coli*. Overexpression of genes encoding QBP27508.1 and AFL47010.1 in *E. coli* BL21(DE3) using the pET21b(+) vector resulted in a significant increase in trimethoprim resistance. The minimum inhibitory concentration (MIC) for trimethoprim was slightly lower than the positive control *dfrA49* gene but significantly higher than that of the control strains harboring empty vectors or overexpressing *folA* (Table 1). Similarly, expression of these genes in *E. coli* TOP10 using the pACYC184 vector also conferred significant trimethoprim resistance. These MIC values were lower than the positive control *dfrA49* gene but significantly higher than those of the control strain with an empty pACYC184 vector (Table 1). Consequently, we designated these genes as *dfrA50* (NG_242637.1, encoding QBP27508.1) and *dfrA51* (NG_242639.1, encoding AFL47010.1), respectively.

**TABLE 1:** Antibiotic susceptibility testing of *E. coli* BL21(DE3) and TOP10 expression of two novel *dfrA* genes (*dfrA50* and *dfrA51*)

Strain, plasmid	MIC (mg/L) of trimethoprim
*E. coli* BL21, pET21b	4 ± 0
*E. coli* BL21, *folA*-pET21b	16 ± 0
*E. coli* BL21, *dfrA49-*pET21b	2,048 ± 0
*E. coli* BL21, *dfrA50*-pET21b	512 ± 0
*E. coli* BL21, *dfrA51-*pET21b	512 ± 0
*E. coli* TOP10, pACYC184	2 ± 0
*E. coli* TOP10, *dfrA49-*pACYC184	2,048 ± 0
*E. coli* TOP10, *dfrA50*-pACYC184	128 ± 0
*E. coli* TOP10, *dfrA51-*pACYC184	256 ± 0

Interestingly, the MIC values of *dfrA50* and *dfrA51* for trimethoprim are lower than those of *dfrA49*. Trimethoprim resistance results from the insensitivity of the DHFR encoded by *dfrA* (12). The reduction in trimethoprim binding of DHFRs is responsible for trimethoprim resistance (15). Previous studies have shown that the MIC value of *dfrA42* to trimethoprim is greater than that of *dfrA43*, and the binding ability of its encoded product DfrA42 to trimethoprim is about four times lower than that of DfrA43 when expressed in the low-copy plasmid pACYC184 (15). This suggests that the resistance level of DfrA to trimethoprim may be negatively correlated with its binding ability to trimethoprim.

To investigate the structural basis underlying the observed differences in resistance, we predicted the three-dimensional structures of DfrA49, DfrA50, and DfrA51 using AlphaFold 3 (16) (Fig. S2A through C). Structural comparisons revealed that DfrA50 and DfrA51 have a high degree of similarity, with a root mean square deviation (RMSD) of 0.287 Å (Fig. S2D). Although DfrA50 and DfrA51 share less than 50% sequence identity with DfrA49, structural homology analysis showed that their overall fold remains highly conserved compared with DfrA49. The RMSD values between DfrA49 and DfrA50/DfrA51 were 0.532 Å and 0.662 Å, respectively (Fig. S2E and F), suggesting a conserved structural framework.

To further explore the potential differences in substrate interactions, we performed molecular docking analysis using AutoDock 1.5.7 to predict the potential binding patterns of trimethoprim with DfrA49, DfrA50, and DfrA51. The predicted binding sites for DfrA50 and DfrA51 were A6, I13, E26 (D26 in DfrA51), and S48, whereas the key residues for DfrA49 were D20, S50, N47, and Y107 (Fig. S3A through C). These findings suggest that although the overall structures are conserved, differences in key binding residues may contribute to variations in trimethoprim resistance.

To experimentally validate this hypothesis, we measured the binding affinities of EcDHFR (encoded by *folA*), DfrA49, DfrA50, and DfrA51 for trimethoprim using isothermal titration calorimetry (ITC). However, despite multiple optimization attempts, DfrA50 rapidly precipitated after purification, preventing its measurement. The K_d_ values for EcDHFR, DfrA49, and DfrA51 were determined to be (7.30 ± 6.60) × 10^−7^ M, (3.29 ± 1.46) × 10^−5^ M, and (6.41 ± 3.63) × 10^−6^ M, respectively (Fig. S4). These findings suggest a negative correlation between MIC values and the binding affinity of the protein for trimethoprim. The lower MIC value of *dfrA51* may be attributed to the stronger binding affinity of its encoded protein for trimethoprim compared with DfrA49. This stronger interaction may more effectively inhibit enzymatic activity, thereby reducing the conversion efficiency of dihydrofolate to tetrahydrofolate and ultimately compromising bacterial growth and resistance.

To evaluate the functional significance of the predicted active site residues, we introduced site-directed mutations into *dfrA50* (A6V, I13A, E26A, S48A) and *dfrA51* (A6V, I13A, D26A, S48A) and expressed the mutant genes in *E. coli* BL21(DE3) to determine their MIC values against trimethoprim. Compared with *E. coli* BL21(DE3) expressing wild-type *dfrA50*, strains carrying the A6V, I13A, and S48A variants exhibited 8-fold, 4-fold, and 4-fold reductions in trimethoprim resistance, respectively, whereas the E26A mutation had no significant effect (Table S2). Similarly, compared with *E. coli* BL21(DE3) expressing wild-type *dfrA51*, strains harboring the A6V, I13A, D26A, and S48A mutations showed 16-fold, 4-fold, 32-fold, and 4-fold reductions in trimethoprim resistance, respectively (Table S2). These findings indicate that residues A6, I13, and S48 are critical for maintaining trimethoprim resistance in both DfrA50 and DfrA51, whereas E26 in DfrA50 does not appear to play a significant role in resistance.

In summary, our structural and biochemical analyses suggest that although DfrA50 and DfrA51 share an overall conserved fold with DfrA49, differences in key binding residues likely contribute to their reduced trimethoprim resistance. The stronger binding affinity of DfrA51 for trimethoprim appears to compromise its enzymatic activity, leading to lower MIC values. Further investigations are required to determine whether similar mechanisms apply to DfrA50, as its binding properties remain unresolved due to protein instability.

### Phylogenetic analysis of these novel DfrA encoded by phage-plasmids

**Fig 2:**
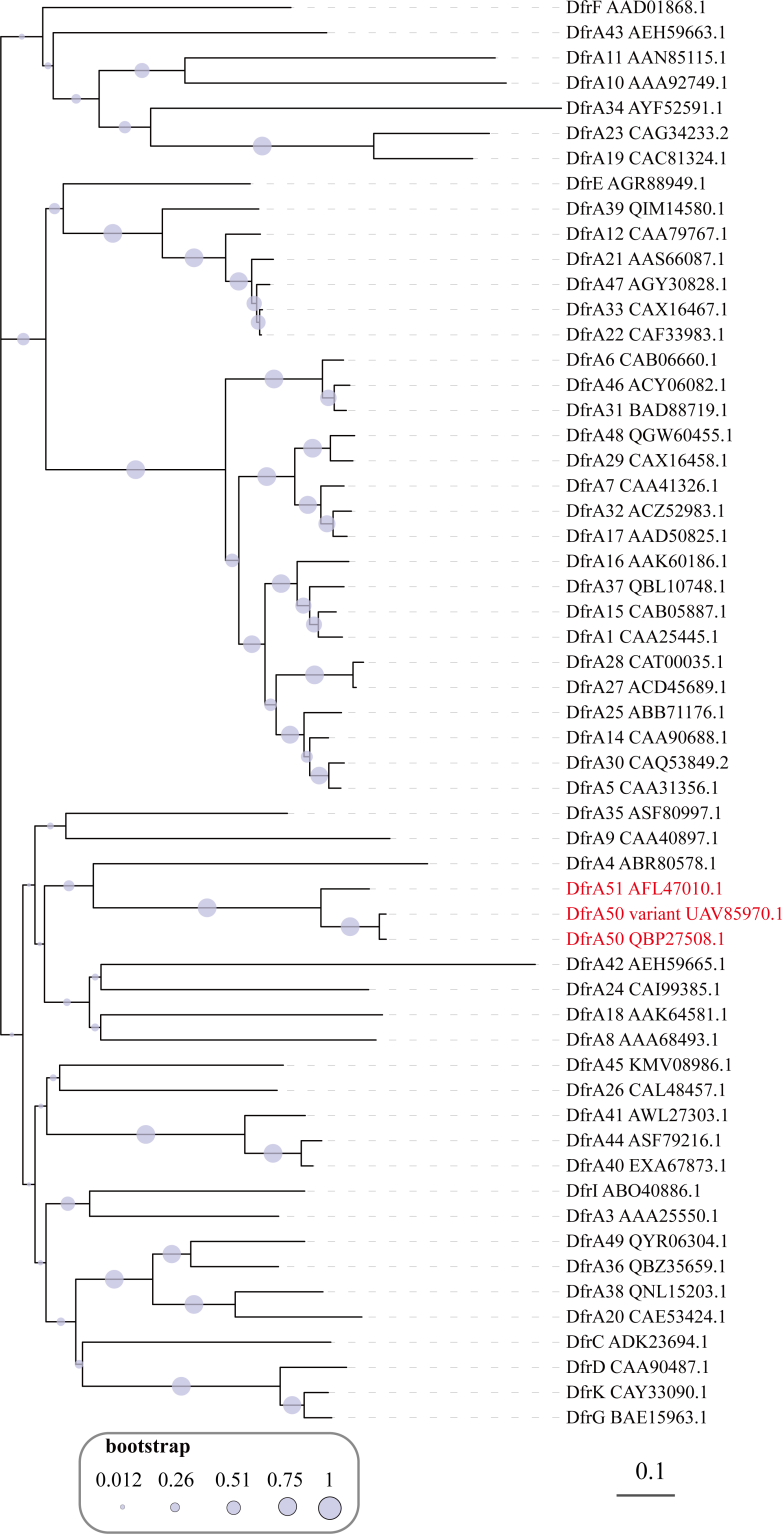
Phylogenetic tree of all reported DfrAs including the three novel DfrAs identified in this work. Phylogenetic analysis was performed with amino acid sequences of all reported DfrAs. The DfrC-K protein sequences were obtained from the NCBI Reference Gene Catalog (https://www.ncbi.nlm.nih.gov/pathogens/refgene), which the amino acid sequences of DfrA1-48 and DfrA49 were retrieved from previous literature (12, 17). The analysis was conducted using MEGA X with 1,000 bootstrap replicates based on the alignment of protein sequences and the neighbor-joining method. The three newly identified DfrAs are highlighted in red font. The scale bar refers to the number of substitutions per site.

Relationships between DfrA50, DfrA51, and previously reported members of the DfrA family were explored by constructing a phylogenetic tree. These DfrAs did not cluster with other reported DfrAs but instead formed a distinct subclade (Fig. 2), suggesting that these DfrAs may have distinct evolutionary origins compared with those previously reported as being derived from plasmids or bacterial chromosomes. The amino acid sequence identity of DfrA50 and DfrA51 with all reported DfrAs from gram-positive and gram-negative bacteria is less than 50%, whereas the amino acid identity between DfrA50 and DfrA51 is 78.98% with 99% coverage, which supports the conclusion of the phylogenetic tree.

### The *dfrA* genes encoded by phage-plasmids are found in various pathogenic bacteria

The presence of phage-plasmids carrying novel *dfrA* genes in host bacteria suggests their potential role in spreading these genes. Phage-plasmids can transmit ARGs via phage infection and plasmid conjugation (8), raising concerns about their potential widespread dissemination, especially among pathogenic bacteria.

**Fig 3:**
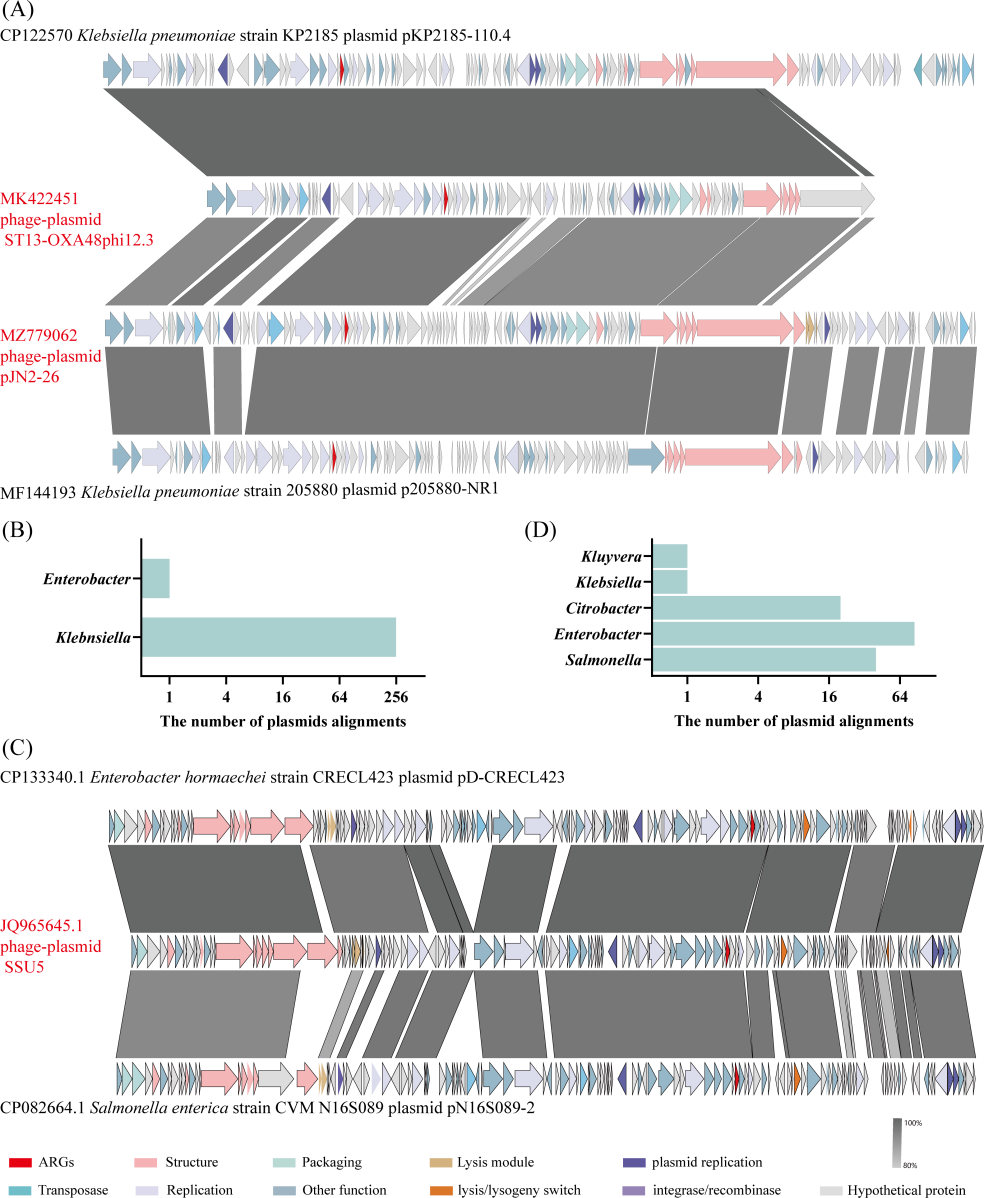
Genomic alignment phage-plasmids with their corresponding bacterial-plasmids containing *dfrA* genes. (**A**) Genomic comparison between the genomes of *Klebsiella* phage-plasmid pJN2-26, *Klebsiella* phage-plasmid ST13-OXA48phi12.3, and some plasmid sequences carried by *K. pneumoniae*. Shading indicates high nucleotide identity between different regions of the sequence as determined by BLASTn analysis, with the depth of gray representing varying levels of identity. (**B**) Abundance analysis on plasmids identified in diverse bacterial species, exhibiting a high degree of identity to *Klebsiella* phage-plasmid ST13-OXA48phi12.3. Each of these plasmids harbors the *dfrA*50 gene. “The number of plasmid alignments” represents the number of instances where phage-plasmid sequences were found to match bacterial plasmid sequences. (**C**) Genomic comparison between the genomes of *Salmonella* phage-plasmid SSU5 and some plasmid sequences carried by *Salmonella* and *Enterobacter* bacteria. (**D**) Abundance analysis on plasmids identified in diverse bacterial species, exhibiting a high degree of similarity to *Salmonella* phage-plasmid SSU5. Notably, each of these plasmids harbors the *dfrA*51 gene. Novel ARGs are represented by red color.

To investigate the distribution of phage-plasmids carrying *dfrA* genes that act as plasmids in bacteria, a BLASTn analysis against the NCBI nt database was conducted, with a criterion of ≥80% coverage and ≥80% identity with whole phage-plasmid genomes. Given the 90% coverage and 97.76% genomic identity between *Klebsiella* phage-plasmid pJN2-26 and *Klebsiella* phage-plasmid ST13-OXA48phi12.3, we focused exclusively on the latter in our investigation. Our findings revealed that the *Klebsiella* phage-plasmid ST13-OXA48phi12.3, carrying *dfrA50*, showed ≥82% coverage and ≥96% identity to plasmid sequences found in many *Klebsiella* strains, except for those in *Enterobacter*, where all these plasmids carried *dfrA50* genes (Fig. 3A and B; Table S3). This suggests that *dfrA50* transmission may primarily occur via infection and lysogenic conversion of phage-plasmid ST13-OXA48phi12.3. Similarly, *Salmonella* phage-plasmid SSU5, carrying *dfrA51*, showed ≥80% coverage and ≥91% identity to plasmid sequences of various bacterial species, including *Salmonella*, *Enterobacter*, and *Citrobacter*, and all of these plasmids carried *dfrA51* genes (Fig. 3C and D; Table S3). However, given the rarity of phages infecting bacteria across different genera and the absence of conjugative transfer genes in the SSU5 genome (18), it is unlikely that *dfrA51* is transmitted via phage infection or conjugation. This implies potential alternative mechanisms for its transmission. Overall, our findings highlight the extensive dissemination of *dfrA50* and *dfrA51*-carrying phage-plasmids among diverse pathogenic bacteria in natural environments, indicating that these bacteria may have developed resistance to trimethoprim. Consequently, exploring alternative drugs for the clinical treatment of infections caused by these bacteria is imperative.

Interestingly, previous studies have found that phage-plasmid pJN2-26 exhibits high nucleotide sequence identity with the *Klebsiella* phage-plasmid ST13-OXA48phi12.3 (90% coverage and 97.76% identity) and the *Salmonella* phage-plasmid SSU5 (74% coverage and 76.75% identity) (19), suggesting that the phage-plasmids ST13-OXA48phi12.3, pJN2-26, and SSU5 may share a common ancestor.

ARGs in plasmids are typically found within integrons or adjacent to insertion sequences, which likely play an important role in the acquisition and dissemination of ARGs (3, 20). However, phages seldom carry these mobile elements (21). Previous studies have shown that ARGs in phage-plasmids are often flanked by mobile elements (8), such as the *bla*_SHV-2_ gene in *Escherichia* phage-plasmid RCS47 (22). Interestingly, our analysis revealed that no mobile elements were located near *dfrA50* and *dfrA51* (Fig. 1B). This suggests that these *dfrA* genes may have originated from ancestral phage-plasmids, rather than being acquired externally during evolution.

In this study, we identified novel functional *dfrA* genes in phage-plasmids, encoding proteins with less than 50% amino acid identity to all previously reported DfrA proteins. Similarly, previous studies have shown that phages often encode novel proteins with no homology to known proteins yet possessing unique functions, such as a unique *de novo* replicative DNA polymerase (23), a novel functional CRISPR-CasΦ system (24). Based on these findings, we hypothesize that phage-plasmid-encoded genes of unknown function may also represent novel antibiotic resistance determinants. Therefore, future research employing high-throughput functional metagenomic screening or structure-based bioinformatics analyses may uncover previously unidentified antibiotic resistance genes.

### Conclusions

In summary, our study unveils that several phages containing plasmid features harbor novel functional *dfrA* genes (*dfrA50* and *dfrA51*), facilitating their widespread dissemination among multiple pathogenic bacteria in the natural environment. Given the ability of these phages to rapidly spread *dfrA* genes through both phage infection and plasmid conjugation pathways, there is an urgent need to enhance monitoring efforts for *dfrA* genes carried by these phages to address their potential threat for antibiotic resistance.

## MATERIALS AND METHODS

### Identification of phages with plasmid features (phage-plasmids) that already act as bacterial plasmids

We collected all 12,443 available phage genome sequences from the NCBI Nucleotide Database (as of November 25, 2021), excluding genomes smaller than 10 kb, as no dsDNA phages have been reported to be smaller than that size (8). We employed a previously described method (7) to identify 1,248 phages exhibiting plasmid features, referred to as phage-plasmids. Briefly, we searched for genes associated with plasmid features in all collected phage genomes using Hidden Markov Models (HMMs) based on protein profiles specific to plasmid replication and partition systems (7). Subsequently, we performed BLASTn searches on the candidate phage genomes against the nonredundant NCBI RefSeq Plasmid Database (25). The selected candidate phage genomes exhibited ≥80% coverage and ≥80% identity with complete plasmid sequences in bacterial genomes. This approach identified 46 phages with plasmid features (phage-plasmids) that function as both phages and bacterial plasmids in natural environments.

### Identification of candidate ARGs

Previous studies have shown that lenient threshold search strategies can unveil novel and unreported ARGs (26). Therefore, we applied these strategies to identify all candidate ARGs in 46 phage-plasmid genomes mentioned above. Specifically, we downloaded all protein sequences encoded by these 46 phage-plasmids and used them as queries in BLASTp analyses to search for ARG sequences against the CARD database (with an E-value threshold of ≤1e^−5^ and query coverage ≥60%) (27). Additionally, we performed HMMScan analyses to search sequences in the RESFAMS ARG database (with an E-value threshold of ≤1e^−5^ and a ≥ 40 score value) (28).

### Construction of plasmids and transformed *E. coli* strains expressing *dfrA* genes

All primers used in this study are listed in Table S4. The two candidate *dfrA*-like genes, *dfrA50* (RefSeq nucleotide accession NG_242637.1) and *dfrA51* (RefSeq nucleotide accession NG_242639.1), along with the positive control *dfrA49* gene (RefSeq nucleotide accession NG_242636.1) (17), underwent codon optimization for expression in *E. coli* (Fig. S5). These optimized genes were synthesized by AuGCT Biotech. These *dfrA* genes (*dfrA50*, *dfrA51*, and *dfrA49*) were then amplified using synthetic genes as templates with Ex Taq DNA polymerase. The *folA* gene, which encodes chromosomal dihydrofolate reductase (RefSeq nucleotide accession NP_414590.1, protein accession AOO72573.1), was amplified using the *E. coli* DH5α genomic DNA (GenBank accession number CP017100) as a template, also with Ex Taq DNA polymerase. Subsequently, these genes (*folA*, *dfrA50*, *dfrA51*, and *dfrA49*) were individually subcloned into the pET21b(+) vector using homologous recombination (Vazyme ClonExpress II One Step Cloning Kit) as described by the manufacturers (Fig. S6A). The correct clones were verified by Sanger sequencing. The resulting recombinant vectors, along with the empty pET21b(+) vector, were extracted from *E. coli* DH5α using the alkaline extraction method described previously (29). The vectors were then transformed into *E. coli* BL21(DE3) cells via chemical transformation (30). Colonies growing on plates containing 100 µg/mL ampicillin were selected, and recombinant strains were identified by colony PCR using the primers T7 and T7 terminator (31).

Simultaneously, *dfrA50*, *dfrA51*, and *dfrA49* genes were fused with the promoter of chloramphenicol resistance gene (*P_cmR_*) from plasmid pACYC184 using the splicing by overlap extension (SOE) PCR method, as described in Fig. S6B. The first step involved generating *P*_cmR_ fusions with the *dfrA* genes, as shown in Fig. S6B. The resulting amplified fusion fragment products were digested with *Xba*I and *Hin*dIII, then individually subcloned into the pACYC184 vector (low copy number) that had been previously digested with the same restriction enzymes. The ligation was carried out using T4 ligase. Correct clones were confirmed by Sanger sequencing, and plasmids were extracted using the alkaline lysis method. Following subcloning, the recombinant plasmids and the empty pACYC184 vector were transformed into *E. coli* TOP10 strain by chemical transformation (30). Colonies growing on plates containing 25 µg/mL chloramphenicol were selected, and recombinant strains were identified by colony PCR using the primers pACYC184-F and pACYC184-R (31).

### Antimicrobial susceptibility testing of two novel *dfrA* genes

To assess the resistance of these recombinant strains to trimethoprim, a broth macrodilution method was employed in accordance with the Clinical and Laboratory Standards Institute (CLSI) guidelines (32). Specifically, these strains were cultured in 2 mL of Mueller-Hinton medium supplemented with different trimethoprim concentrations (ranging from 0.5 to 2,048 mg/L in doubling concentrations), and growth or absence of growth was determined by measuring the turbidity of the cultures. All BL21(DE3) strains were supplemented with a final concentration of 0.1 mmol/L isopropyl β-D-1-thiogalactopyranoside (IPTG) to induce the T7 promoter in pET21b(+) expressing *folA*/*dfrA* genes. The strain expressing DfrA49 served as a positive control, whereas strains containing the empty pET21b(+) or pACYC184 vectors and overexpressing the *folA* gene in pET21b(+) functioned as negative controls. These experiments were conducted in triplicate.

### Protein structure prediction and molecular docking with trimethoprim

The three-dimensional structures of DfrA49, DfrA50, and DfrA51 were predicted using the AlphaFold 3 web server (16). The structure of trimethoprim was obtained from the PubChem database in SDF format and converted to PDB format using OpenBabel 3.1.1.

For molecular docking, AutoDock 1.5.7 was used. First, the target protein structures were preprocessed by removing water molecules and adding hydrogen atoms and then saved in PDBQT format. The trimethoprim ligand was also preprocessed using AutoDock Tools by adding hydrogen atoms, defining rotatable bonds, and saving it in PDBQ format for docking simulations.

Docking was performed using AutoDock 1.5.7 software. The "autogrid" function was used to generate grid maps for the selected protein, defining the docking space for ligand binding. The entire protein was set as the docking grid box. The genetic algorithm was selected as the search method, with 50 independent docking runs. The Lamarckian genetic algorithm was used for energy minimization.

The docking results generated 50 binding conformations. The conformation with the lowest binding energy and highest clustering frequency was considered the most probable binding mode and was selected for visualization and analysis using PyMOL 2.4.

### Expression and purification of EcDHFR and DfrA proteins

To express the EcDHFR and DfrA proteins, the *E. coli* BL21(DE3) strain harboring pET21b (+) plasmids for *folA*, *dfrA49*, *dfrA50*, or *dfrA51* was cultured in LB medium. Once the culture reached mid-log phase (OD600 ≈ 0.6–0.8), the expression was induced with 0.2 mM isopropyl β-D-1-thiogalactopyranoside (IPTG) and allowed to proceed overnight at 16 ℃. Afterward, cells were harvested by centrifugation at 8,000 × *g* for 10 min at 4 ℃, resuspended in 25 mL of PBS buffer, and disrupted on ice using a high-pressure cell disruptor (JN-MiniPro). The lysate was centrifuged at 10,000 × *g* for 60 min at 4°C to remove cell debris, and the supernatant containing the soluble proteins was collected. The soluble fraction was applied to a pre-equilibrated Ni-NTA affinity column (Qiagen). Non-specifically bound proteins were washed away with a PBS buffer containing 150 mM NaCl and 60 mM imidazole. Target proteins were eluted with a buffer containing PBS, 150 mM NaCl, and 300 mM imidazole. Protein purity was assessed via SDS-PAGE, and the eluted fractions were desalted into PBS using a HiTrap Desalting Column. The purified proteins were stored at −80 ℃ for future use.

### Isothermal titration calorimetry assay

To evaluate the interactions between trimethoprim and EcDHFR, DfrA49, and DfrA51, isothermal titration calorimetry (ITC) experiments were performed using the Auto iTC200 system. All titrations were conducted at 25 ℃ with continuous stirring at 750 rpm in PBS buffer containing 1% dimethyl sulfoxide (DMSO). To account for heat effects due to dilution, controlled titrations of ligands into buffer were performed. The protein concentration was maintained between 15-30 μM. Before titration, proteins were pre-incubated on ice for 1 h at 4 ℃ with a 10-fold molar excess of nicotinamide adenine dinucleotide phosphate (NADPH). DMSO was then added to adjust the buffer to 1% DMSO in PBS. Trimethoprim was prepared at a concentration approximately 10 times higher than that of the protein and dissolved in PBS containing 1% DMSO. All ITC data were analyzed using MicroCal LLC ITC200 software.

### Site-directed mutagenesis of *dfrA50* and *dfrA51*

Site-directed mutagenesis of *dfrA50* and *dfrA51* was performed using the SOE PCR method, utilizing ExTaq DNA polymerase (TaKaRa) as described by the manufacturer. The primers used for mutagenesis are listed in Table S4. Briefly, two separate PCR reactions were performed with wild-type *dfrA* genes as templates to amplify the 5’ and 3’ fragments containing the desired mutations. The resulting overlapping PCR fragments were then used as templates for a second round of PCR to generate full-length mutated *dfrA* sequences. The final PCR products were gel-purified, and the recombinant plasmids were subcloned into the pET21b(+) vector using the Vazyme ClonExpress II One Step Cloning Kit. The correct clones were verified by Sanger sequencing.

The resulting recombinant vectors were extracted from *E. coli* DH5α using the alkaline extraction method. The plasmids were then transformed into *E. coli* BL21(DE3) cells via chemical transformation. Colonies growing on plates containing 100 µg/mL ampicillin were selected, and recombinant strains were identified by colony PCR using the primers T7 and T7 terminator.

### Phylogenetic analysis of these DfrA proteins encoded by phage-plasmids

To determine the evolutionary relationship between the newly identified DfrA proteins and the reported DfrA variants, we collected amino acid sequences for reported DfrA proteins from the GenBank database, including DfrA1-49 from gram-negative bacteria (12, 17) and DfrC-K from gram-positive bacteria (available at https://www.ncbi.nlm.nih.gov/pathogens/refgene). We performed multiple sequence alignment of these sequences using ClustalX2 (33) and conducted phylogenetic analyses using MEGA X (34), employing the neighbor-joining method with 1,000 bootstrap replicates. The resulting phylogenetic tree was visualized using iTOL (35).

### Comprehensive analysis of mobile elements in phage-plasmids

All protein sequences encoded by the phage-plasmids were downloaded from the NCBI database. Insertion sequences (IS elements) were identified using BLASTp against the ISfinder database (36), using a query size >150 amino acids, an E-value <1 × 10^−30^, and an identity >40%, as described by Ettenne et al. (37). Conjugative elements were detected using ConjScan software (38), with an E-value <0.001 and sequence coverage >50%. Integrons were detected using IntegronFinder software (39) with default parameters. Transposases were identified through Batch CD-Search (https://www.ncbi.nlm.nih.gov/Structure/bwrpsb/bwrpsb.cgi). Mobile genetic element (MGE)-related protein candidates were further validated using the Conserved Domain Database (CDD) from NCBI.

## Data Availability

Nucleotide and protein sequences for all *dfrA* genes included in this study have been registered in the NCBI Reference Gene Catalog under the following accession numbers: *dfrA49* (NG_242636.1, WP_038694170.1, or QYR06304.1), *dfrA50* (NG_242637.1, WP_040203768.1, or QBP27508.1), and *dfrA51* (NG_242639.1, WP_000781812.1, or AFL47010.1).

## SUPPLEMENTAL MATERIAL

Supplemental material aac.01695-24-s0001.pdf Fig. S1 to S6; Tables S1, S2, and S4.

Table S3 aac.01695-24-s0002.xlsx Overview of the identified bacterial plasmids.

ASM does not own the copyrights to Supplemental Material that may be linked to, or accessed through, an article. The authors have granted ASM a non-exclusive, world-wide license to publish the Supplemental Material files. Please contact the corresponding author directly for reuse.
