# Supplementary material for: Two novel trimethoprim resistance genes, dfra50 and dfra51, identified in phage-plasmids
Source: Antimicrob Agents Chemother. 2025 Jun 12;69(7):e01695-24. doi: 10.1128/aac.01695-24 (PMC12217456; doi:10.1128/aac.01695-24)
Supplement: Supplemental material — Fig. S1 to S6; Tables S1, S2, and S4. [file aac.01695-24-s0001.pdf]

Supplementary Materials for

Two novel trimethoprim resistance genes, *dfrA50* and *dfrA51*, identified in phages-plasmids

Kai Wang,[a,b] Jikai Xu,[a,b] Xiaowei Lu,[a,b] Pan Yin,[a,b] Li Chen,[a,b] Ziwei Zhao,[a,b] Alejandra Bravo,[d] Mario Soberón,[d] Jinshui Zheng,[a,c] Ming Sun,[a,b] Donghai Peng,[a,b#]

[a]National Key Laboratory of Agricultural Microbiology, Huazhong Agricultural University, Wuhan 430070, Hubei, China

[b]College of Life Science and Technology, Huazhong Agricultural University, Wuhan 430070, China.

[c]College of Informatics, Huazhong Agricultural University, Wuhan, China.

[d]Instituto de Biotecnología, Universidad Nacional Autónoma de México, Apdo. postal 510-3, Cuernavaca, 62250 Morelos, Mexico.

Running Head**:** Phage-plasmids carry novel *dfrA* genes

#Address correspondence to Donghai Peng, donghaipeng@mail.hzau.edu.cn. TEL: 86-27-87280670. FAX: 027-87280670.

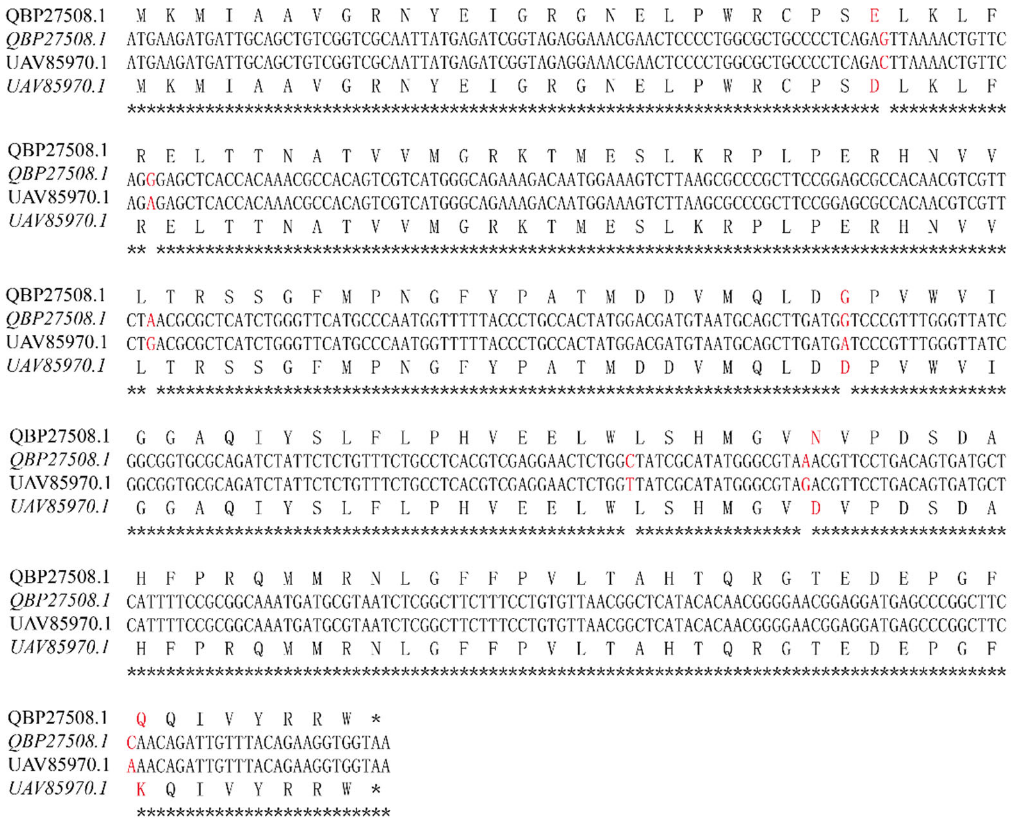

Figure S1. Multiple sequence alignment of the nucleotide sequences of the genes encoding QBP27508.1 and UAV85970.1 and the amino acid sequences of their encoded products.

Red indicates different nucleotides or amino acids.

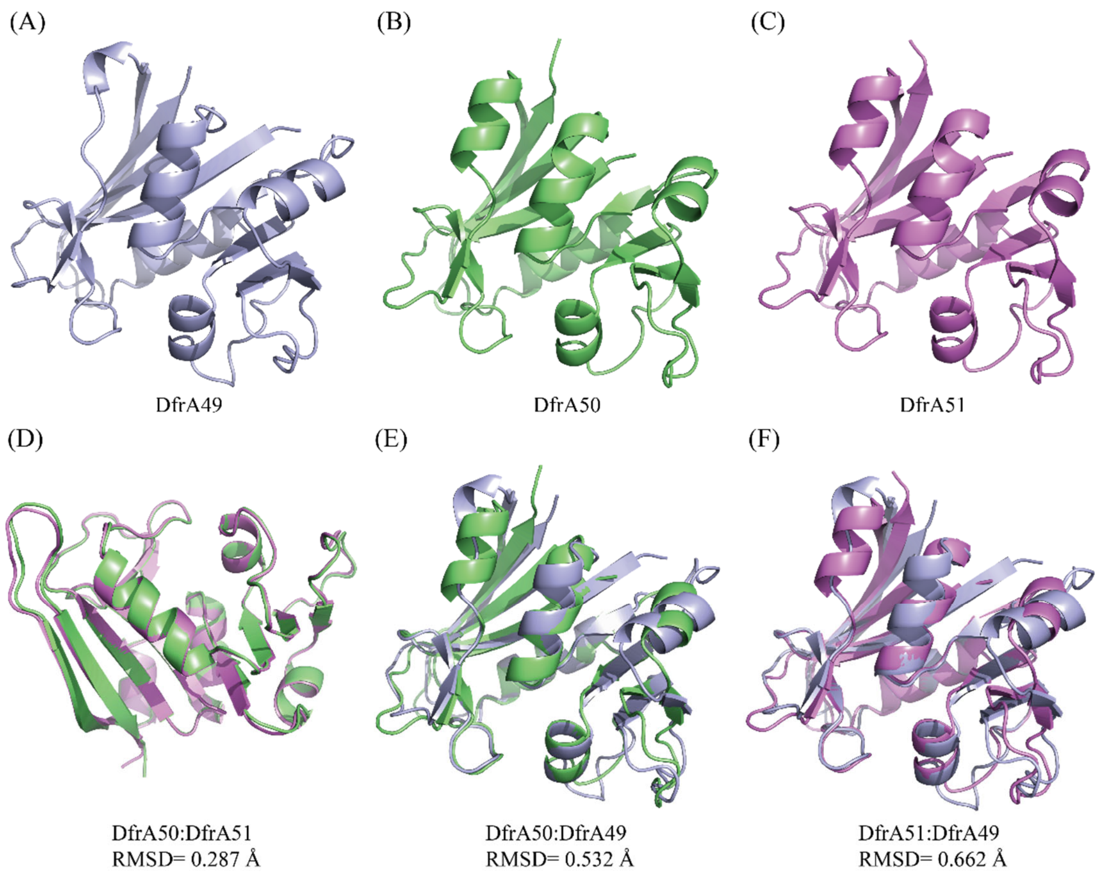

Figure S2. Structural comparison of DfrA49, DfrA50, and DfrA51.

(A-C) Predicted three-dimensional structures of DfrA49, DfrA50, and DfrA51 using AlphaFold 3.

(D) Structural alignment of DfrA50 and DfrA51, showing a high degree of similarity with an RMSD of 0.287 Å.

(E–F) Structural comparisons of DfrA49 with DfrA50 and DfrA51, with RMSD values of 0.532 Å and 0.662 Å, respectively.

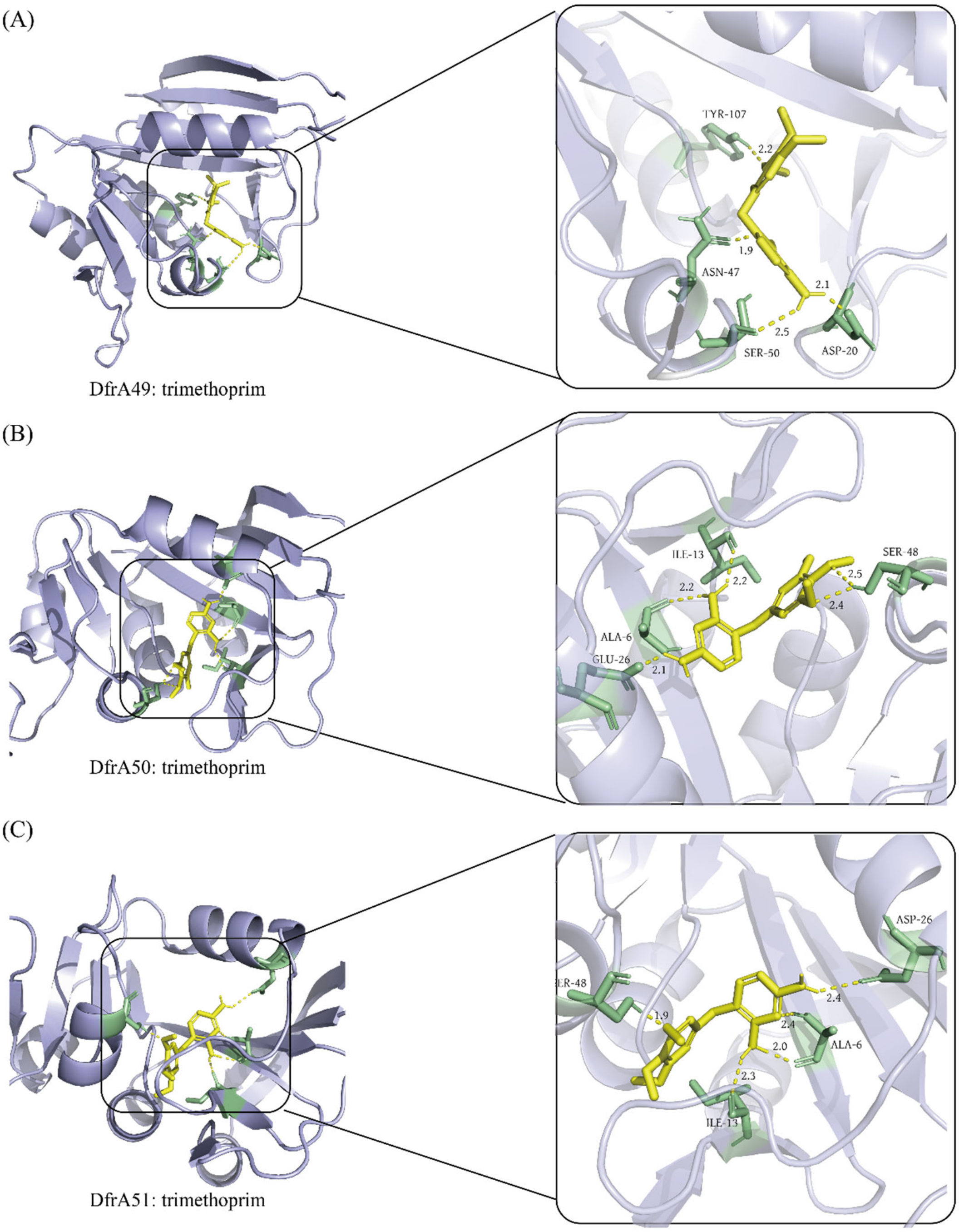

Figure S3. Molecular docking analysis of trimethoprim binding to DfrA49, DfrA50, and DfrA51.

(A) Predicted binding interactions of trimethoprim with DfrA49, highlighting key residues D20, S50, N47, and Y107. **(B)** Predicted binding sites for trimethoprim with DfrA49, highlighting key residues A6, I13, E26, and S48. **(C)** Predicted binding sites for trimethoprim with DfrA49, highlighting key residues A6, I13, D26, and S48. In all panels, the trimethoprim is shown in yellow, interacting residues are displayed in palegreen, and the overall protein structure is represented in light blue. Hydrogen bonds

between trimethoprim and binding site residues are indicated by yellow dashed lines. Molecular docking was performed using AutoDock 1.5.7.

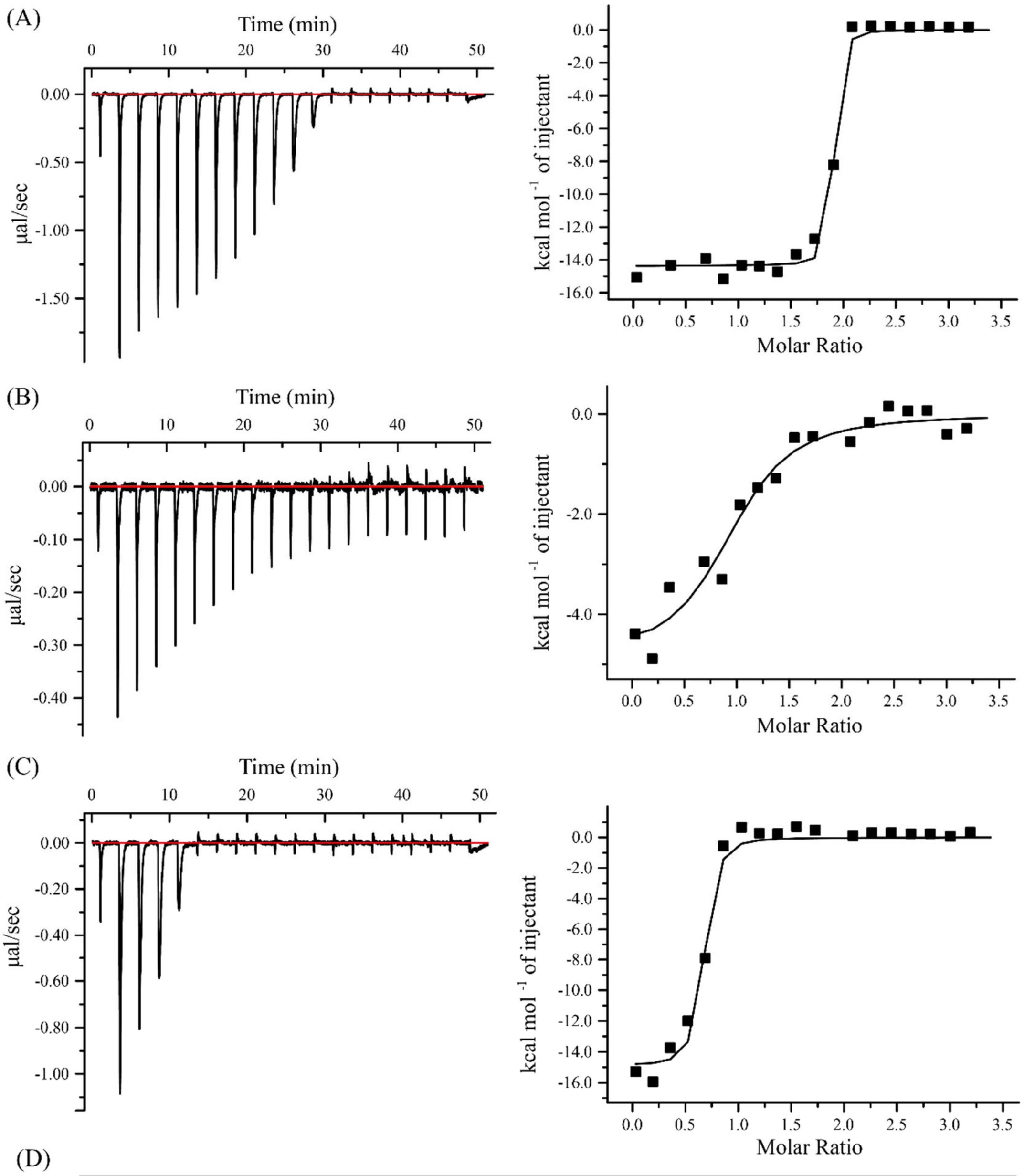

(D)

Sample	EcDHFR	DfrA49	DfrA51
K_d (M)	$(7.30 \pm 6.60) \times 10^{-7}$	$(3.29 \pm 1.46) \times 10^{-5}$	$(6.41 \pm 3.63) \times 10^{-6}$

Figure S4. Isothermal titration calorimetry (ITC) analysis of trimethoprim binding to EcDHFR, DfrA49, and DfrA51.

(A) ITC results for trimethoprim binding to the EcDHFR: NADPH complex. **(B)** ITC results for trimethoprim binding to the DfrA49: NADPH complex. **(C)** ITC results for trimethoprim binding to the DfrA51: NADPH complex. **(D)** Comparison of K_d values for trimethoprim binding to EcDHFR, DfrA49, and DfrA51. Each ITC panel (A–C) displays the raw heat signal (left) and the corresponding binding isotherm (right).

(A)

```
amino acid         M  K  V  A  L  I  V  A  V  D  Q  Q  F  G  I  G  K  N  N  D  L  M  W  H  L  P  A  D  M  K 
dfrA49            ATGAAAGTAGCATTGATTGTTGCTGTTGATCAGCAATTCGGTATTGGAAAGAACAATGATTTGATGTGGCATTTGCCTGCAGATATGAAA
dfrA49_optimized  ATGAAGGTGGCACTGATTGTGGCAGTTGATCAGCAGTTTGGCATTGGCAAAAATAATGATCTGATGTGGCACCTGCCGGCAGATATGAAA
                  ***** ** *** ******* ** *********** ** ** ***** ** ** ****** **********  **** ************

amino acid         F  F  K  E  T  T  T  G  H  I  V  V  T  G  R  K  N  Y  D  S  I  P  E  R  F  R  P  L  P  N 
dfrA49            TTTTTCAAAGAAACAACCACGGGACATATTGTGGTTACAGGTAGAAAAAACTACGATTCCATTCCGGAACGTTTTCGGCCGTTGCCCAAC
dfrA49_optimized  TTTTTCAAAGAAACCACCACCGGCCATATTGTGGTTACCGGCCGTAAAAATTATGATAGTATCCCGGAACGTTTCCGCCCGCTGCCTAAT
                  ************** ***** ** ************** **  * ***** ** ***   ** *********** ** *** **** ** 

amino acid         R  E  N  A  V  L  T  R  N  T  E  Y  H  A  P  G  A  V  V  F  S  S  L  E  S  C  L  D  H  Y 
dfrA49            CGCGAGAATGCGGTCTTAACACGCAATACCGAATATCATGCTCCTGGAGCAGTTGTTTTTCTTCCTTGGAATCCTGTTTGGATCATTAT
dfrA49_optimized  CGTGAAAATGCAGTGCTGACCCGCAATACCGAATATCATGCACCGGGTGCAGTGGTGTTTAGTAGTCTGGAAAGTTGTCTGGATCATTAT
                  ** ** ***** **   * ** ******************* ** ** ***** ** ***  *    *****   *** ***********

amino acid         K  N  E  V  E  R  T  V  F  I  I  G  G  G  Q  I  Y  R  E  A  L  A  L  D  C  V  Q  E  M  F 
dfrA49            AAAAATGAAGTGGAACGAACCGTTTTCATAATTGGAGGCGGACAAATTTACCGAGAAGCTTTAGCGCTTGATTGTGTTCAAGAGATGTTT
dfrA49_optimized  AAGAACGAGGTGGAACGCACCGTGTTTATTATTGGTGGTGGTCAGATCTATCGCGAAGCCCTGGCCCTGGATTGTGTGCAAGAAATGTTT
                  ** ** ** ******** ***** ** ** ***** ** ** ** ** ** ** *****  * ** ** ******** ***** ******

amino acid         I  T  H  V  Q  G  E  F  G  A  D  T  F  F  P  K  F  E  A  V  A  W  N  V  E  T  V  A  T  Q 
dfrA49            ATTACCCATGTGCAGGGCGAATTTGGTGCAGATACCTTCTTCCCGAAATTCGAAGCTGTCGCTTGGAATGTTGAAACGGTAGCAACCCAA
dfrA49_optimized  ATTACCCACGTTCAGGGTGAATTTGGTGCCGATACCTTTTTCCCGAAATTTGAAGCAGTGGCCTGGAATGTTGAAACCGTGGCCACCCAG
                  ******** ** ***** *********** ******** *********** ***** ** ** ************** ** ** ***** 

amino acid         A  V  D  E  K  N  A  Y  A  F  E  V  K  R  Y  W  R  * 
dfrA49            GCAGTGGATGAGAAAAATGCCTATGCGTTTGAAGTGAAAAGGTATTGGAGGTAA
dfrA49_optimized  GCAGTTGATGAAAAAAATGCCTATGCATTCGAGGTTAAGCGCTATTGGCGCTAA
                  ***** ***** ************** ** ** **  * ****** * ***
```

(B)

```
amino acid         M  K  M  I  A  A  V  G  R  N  Y  E  I  G  R  G  N  E  L  P  W  R  C  P  S  E  L  K  L  F 
dfrA50            ATGAAGATGATTGCAGCTGTCGGTCGCAATTATGAGATCGGTAGAGGAAACGAACTCCCCTGGCGCTGCCCCTCAGAGTTAAAACTGTTC
dfrA50_optimized  ATGAAGATGATCGCCGCAGTTGGCCGTAATTATGAAATTGGTCGTGGTAATGAACTGCCGTGGCGCTGCCCGAGCGAACTGAAACTGTTT
                  *********** ** ** ** ** ** ******** ** *** * ** ** ***** ** ***********    **  * ******** 

amino acid         R  E  L  T  T  N  A  T  V  V  M  G  R  K  T  M  E  S  L  K  R  P  L  P  E  R  H  N  V  V 
dfrA50            AGGGAGCTCACCACAAACGCCACAGTCGTCATGGGCAGAAAGACAATGGAAAGTCTTAAGCGCCCGCTTCCGGAGCGCCACAACGTCGTT
dfrA50_optimized  CGTGAACTGACCACCAATGCAACCGTGGTTATGGGTCGTAAAACCATGGAAAGTCTGAAACGTCCGCTGCCGGAACGTCATAATGTTGTG
                   * ** ** ***** ** ** ** ** ** *****  * ** ** *********** ** ** ***** ***** ** ** ** ** ** 

amino acid         L  T  R  S  S  G  F  M  P  N  G  F  Y  P  A  T  M  D  D  V  M  Q  L  D  G  P  V  W  V  I 
dfrA50            CTAACGCGCTCATCTGGGTTCATGCCCAATGGTTTTTACCCTGCCACTATGGACGATGTAATGCAGCTTGATGGTCCCGTTTGGGTTATC
dfrA50_optimized  CTGACCCGTAGTAGTGGCTTTATGCCGAATGGTTTTTATCCGGCCACCATGGATGATGTGATGCAGCTGGATGGTCCGGTTTGGGTTATT
                  ** ** **      *** ** ***** *********** ** ***** ***** ***** ******** ******** *********** 

amino acid         G  G  A  Q  I  Y  S  L  F  L  P  H  V  E  E  L  W  L  S  H  M  G  V  N  V  P  D  S  D  A 
dfrA50            GGCGGTGCGCAGATCTATTCTCTGTTTCTGCCTCACGTCGAGGAACTCTGGCTATCGCATATGGGCGTAAACGTTCCTGACAGTGATGCT
dfrA50_optimized  GGCGGTGCCCAGATCTATAGCCTGTTTCTGCCGCATGTTGAAGAACTGTGGCTGAGTCATATGGGCGTTAATGTTCCGGATAGTGATGCC
                  ******** *********   *********** ** ** ** ***** *****    *********** ** ***** ** ******** 

amino acid         H  F  P  R  Q  M  M  R  N  L  G  F  F  P  V  L  T  A  H  T  Q  R  G  T  E  D  E  P  G  F 
dfrA50            CATTTTCCGCGGCAAATGATGCGTAATCTCGGCTTCTTTCCTGTGTTAACGGCTCATACACAACGGGGAACGGAGGATGAGCCCGGCTTC
dfrA50_optimized  CATTTTCCGCGCCAGATGATGCGCAATCTGGGTTTCTTTCCGGTGCTGACCGCCCATACCCAGCGTGGTACCGAAGATGAACCGGGTTTT
                  *********** ** ******** ***** ** ******** *** * ** ** ***** ** ** ** ** ** ***** ** ** ** 

amino acid         Q  Q  I  V  Y  R  R  W  * 
dfrA50            CAACAGATTGTTTACAGAAGGTGGTAA
dfrA50_optimized  CAGCAGATTGTTTATCGTCGTTGGTAA
                  ** ***********  *  * ******
```

(C)

```
amino acid         M  K  M  I  A  A  V  G  R  N  Y  E  I  G  I  G  N  E  L  P  W  R  C  P  T  D  L  K  L  F 
dfrA51            ATGAAGATGATTGCAGCTGTCGGCCGCAACTATGAAATCGGCATTGGCAACGAACTTCCCTGGCGTTGCCCGACCGATCTGAAACTGTTC
dfrA51_optimized  ATGAAGATGATCGCAGCAGTGGGCCGTAATTATGAAATTGGTATTGGTAACGAGCTGCCGTGGCGCTGCCCTACCGACCTGAAGTTATTC
                  *********** ***** ** ***** ** ******** ** ***** ***** ** ** ***** ***** ***** *****   * ***

amino acid         K  Q  L  T  K  N  A  T  V  V  M  G  R  K  T  M  E  S  L  K  R  P  L  P  E  R  H  N  L  V 
dfrA51            AAACAACTCACCAAAAACGCCACTGTCGTGATGGGACGTAAGACGATGGAAAGTCTTAAACGCCCGCTACCAGAGCGCCATAACCTCGTT
dfrA51_optimized  AAACAGCTGACCAAAAACGCCACCGTGGTTATGGGCCGCAAAACCATGGAAAGTCTGAAACGTCCGCTGCCGGAACGCCATAATCTGGTG
                  ***** ** ************** ** ** ***** ** ** ** *********** ***** ***** ** ** ******** ** ** 

amino acid         L  T  R  S  R  G  Y  I  P  N  G  F  Y  P  A  G  I  D  D  V  L  R  L  P  D  P  V  W  V  I 
dfrA51            TTGACGCGCTCTCGTGGCTATATCCCCAATGGTTTCTACCCCGCTGGCATCGATGACGTTTTGAGACTACCAGATCCGGTCTGGGTGATT
dfrA51_optimized  CTGACCCGTAGCCGCGGCTATATTCCGAATGGTTTTTATCCGGCCGGCATTGATGATGTTCTGCGCCTGCCGGATCCGGTGTGGGTTATT
                   **** **    ** ******** ** ******** ** ** ** ***** ***** *** ** * ** ** ******** ***** ***

amino acid         G  G  G  Q  I  Y  S  L  F  M  P  H  V  E  E  I  W  L  S  H  I  G  V  D  V  P  G  A  D  A 
dfrA51            GGTGGCGGGCAGATTTACTCGCTCTTTATGCCACACGTTGAAGAGATCTGGCTGTCGCATATTGGCGTGGATGTGCCGGGCGCGGATGCG
dfrA51_optimized  GGTGGTGGTCAGATCTATAGCCTGTTTATGCCGCATGTTGAAGAAATTTGGCTGAGCCATATTGGTGTGGATGTTCCGGGTGCAGATGCC
                  ***** ** ***** **    ** ******** ** ******** ** ******   ******** ******** ***** ** ***** 

amino acid         F  F  P  A  P  M  M  R  S  L  G  F  V  P  V  E  T  A  Y  T  Q  R  A  N  E  D  E  P  G  F 
dfrA51            TTCTTCCCGGCGCCAATGATGCGTTCTTTAGGGTTCGTACCAGTCGAAACGGCTTATACCCAACGTGCAAATGAGGATGAGCCTGGCTTT
dfrA51_optimized  TTTTTCCCGGCCCCTATGATGCGCAGCCTGGGTTTTGTGCCGGTGGAAACCGCATATACCCAGCGTGCCAATGAAGATGAACCGGGTTTT
                  ** ******** ** ********     * ** ** ** ** ** ***** ** ******** ***** ***** ***** ** ** ***

amino acid         L  Q  I  V  Y  R  R  S  * 
dfrA51            TTGCAGATCGTATACAGAAGGTCGTAA
dfrA51_optimized  CTGCAGATTGTTTATCGTCGCAGTTAA
                   ******* ** **  *  *    ***
```

Figure S5. Nucleotide sequences of the *dfrA* genes before and after optimization, along with their encoded amino acid sequences, were used in this study.

Panels (A), (B) and (C) represent the nucleotide sequences of *dfrA49*, *dfrA50* and *dfrA51* before and after optimization, along with their respective encoded amino acid sequences.

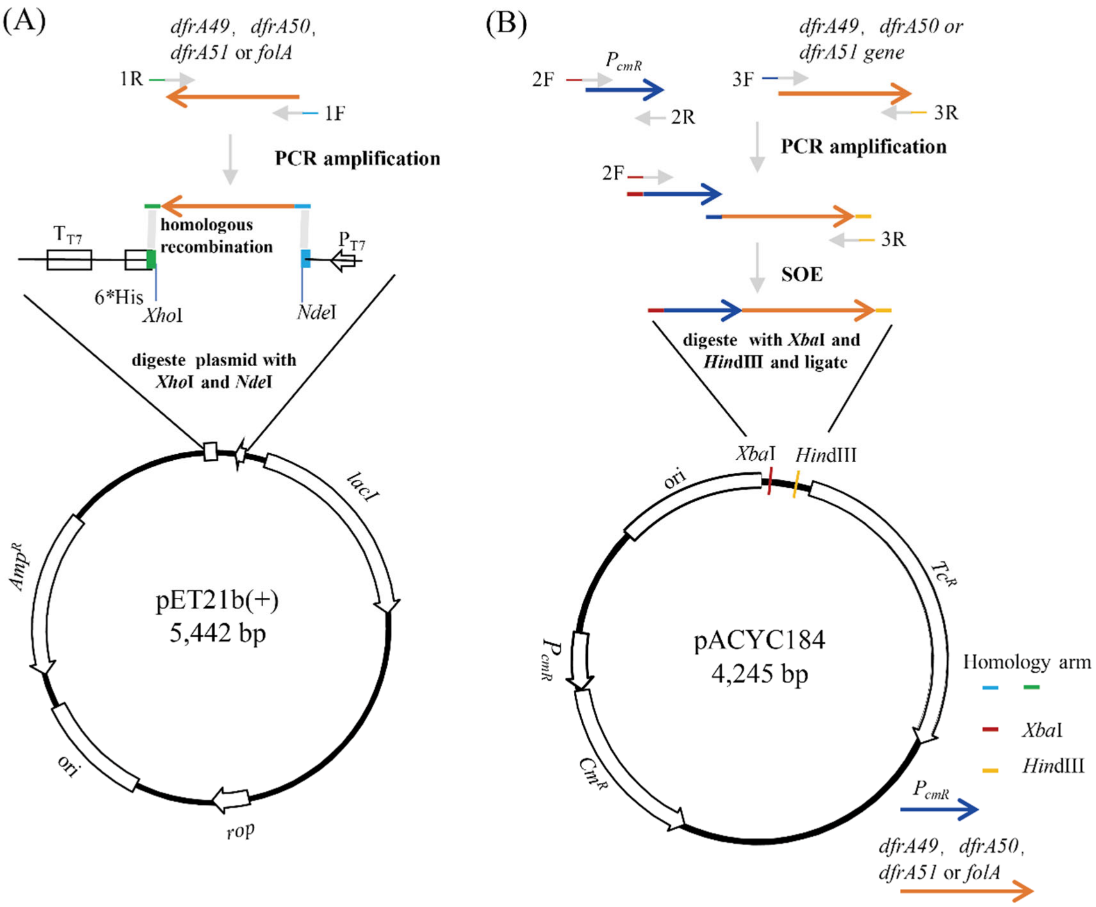

Figure S6. Schematic representation of the amplification and cloning procedures for *dfrA* and *folA* genes.

(**A**) Cloning scheme of *dfrA* and *folA* genes into pET21b(+) vector. *dfrA* and *folA* were amplified using Ex Taq DNA polymerase and recombined with *Nde*I/*Xho*I-digested pET21b(+) using the ClonExpress II One Step Cloning Kit (Vazyme). (**B**) Cloning scheme of *dfrA* genes into pACYC184. The P_{cmR} promoter and *dfrA49*, *dfrA50*, *dfrA51* genes were amplified with Ex Taq DNA polymerase. Fusion fragments of P_{cmR} and *dfrA* genes were generated via splicing by overlap extension (SOE) PCR and digested with *Xba*I/*Hin*dIII. These fragments were then ligated into pACYC184, which had been digested with the same restriction enzymes, using T4 ligase to construct the recombinant plasmids.

Table S1. List of 7 ARGs identified in phage-plasmids

Query protein ID	Search strategies	GenBank accession	Phage-plasmid name	Subject ID	Coverage (Blastp) (%)	Identity (Blastp) (%)	Score (Hmmscan)	e-value	AMR Gene Family	Class of ARGs
BAE47804.1	hmmscan	AP008983	*Clostridium* phage-plasmid c-st	Lactamase_B			58	1.1E-17	PF00753.22 Metallo-beta-lactamase superfamily	MBLs
BAE47805.1	hmmscan	AP008983	*Clostridium* phage-plasmid c-st	Lactamase_B			56.3	3.5E-17	PF00753.22 Metallo-beta-lactamase superfamily	MBLs
AAQ07483.1	Blastp+Hmm scan	AF503408	*Enterobacteri*a phage-plasmid P7	gb|CAD09800.1|ARO:3000873|TEM-1	100	100		0	$bla_{\text{TEM-1}}$	SBLs
AFL47010.1	Blastp+Hmm scan	JQ965645	*Salmonella* phage-plasmid SSU5	gb|BAE15963.1|ARO:3002868|DfrG	77	40.16		2E-29	*dfrA*	DfrA
AMR59984.1	Blastp+Hmm scan	KU760857	*Salmonella* phage-plasmid SJ46	gb|WP_063860005.1|ARO:3005597|CTX-M-27	100	100		0	$bla_{\text{CTX-M-27}}$	SBLs
QBP27508.1	Blastp+Hmm scan	MK422451	*Klebsiella* phage-plasmid ST13-OXA48phi12.3	gb|CAP69659.1|ARO:3003017|DfrA21	89	38.78		4E-30	*dfrA*	DfrA
UAV85970.1	Blastp+Hmm scan	MZ779062	*Klebsiella* phage-plasmid pJN2-26	gb|CAP69659.1|ARO:3003017|DfrA21	89	38.1		7E-30	*dfrA*	DfrA

Table S2. Antibiotic susceptibility testing of *E. coli* BL21 strains expressing mutants of two novel *dfrA* genes (*dfrA50* and *dfrA51*) against trimethoprim.

**Strain, plasmid**	**MIC (mg/L) of Trimethoprim**
E. coli BL21, A6V-*dfrA50*-pET21b	64±0
E. coli BL21, I13A-*dfrA50*-pET21b	128±0
E. coli BL21, E26A-*dfrA50*-pET21b	512±0
E. coli BL21, S48A-*dfrA50*-pET21b	128±0
E. coli BL21, A6V-*dfrA51*-pET21b	32±0
E. coli BL21, I13A-*dfrA51*-pET21b	128±0
E. coli BL21, D26A-*dfrA51*-pET21b	16±0
E. coli BL21, S48A-*dfrA51*-pET21b	128±0

Table S3. Overview of the identified bacterial plasmids that share high nucleotide sequence identity to the phage-plasmids ST13-OXA48phi12.3 carrying *dfrA50*.

separate file.

Table S4. List of primers used in this study.

Primers	Sequence (5'-3')	Functions
P_{cmR}-XbaI-F	CTAGTCTAGATTGCGCCGAATAAATACC	To amplify the promoter of chloramphenicol resistance gene using pACYC184 as template
P_{cmR}-soe-R	TTTAGCTTCCTTAGCTCCTG	
dfrA49-P_{cmR}-F	CAGGAGCTAAGGAAGCTAAAATGAAGGTGGCACTGATTG	To amplify the *dfrA49* gene, and to use the amplified product of P_{cmR} and *dfrA49* as templates to generate the fusion fragment of P_{cmR} with *dfrA49*, by using P_{cmR}-XbaI-F and *dfrA49*-P_{cmR}-HindIII-R primers.
dfrA49-P_{cmR}-HindIII-R	CCCAAGCTTTTAGCGCCAATAGCGCTTAAC	
dfrA50-P_{cmR}-F	CAGGAGCTAAGGAAGCTAAAATGAAGATGATCGCCGCAG	To amplify the *dfrA50* gene, and to use the amplified product of P_{cmR} and *dfrA50* as templates to generate the fusion fragment of P_{cmR} with *dfrA50*, by using P_{cmR}-XbaI-F and *dfrA50*-P_{cmR}-HindIII-R primers.
dfrA50-P_{cmR}-HindIII-R	CCCAAGCTTTTACCAACGACGATAAAC	
dfrA51-P_{cmR}-F	CAGGAGCTAAGGAAGCTAAAATGAAGATGATCGCAGCAG	To amplify the *dfrA51* gene, and to use the amplified product of P_{cmR} and *dfrA51* as templates to generate the fusion fragment of P_{cmR} with *dfrA51*, by using P_{cmR}-XbaI-F and *dfrA51*-P_{cmR}-HindIII-R primers.
dfrA51-P_{cmR}-HindIII-R	CCCAAGCTTTTAACTGCGACGATAAAC	
folA-pET21b-F	AAGAAGGAGATATACATATGATCAGTCTGATTGCGGCG	To amplify the *folA* gene for ligation into pET21b(+)
folA-pET21b-R	TGGTGATGGTGATGCTCGAGCCGCCGCTCCAGAATCTCAAAG	
dfrA49-pET21b-F	AAGAAGGAGATATACATATGAAGGTGGCACTGATTGTG	To amplify the *dfrA49* gene for ligation into pET21b(+)
dfrA49-pET21b-R	TGGTGATGGTGATGCTCGAGGCGCCAATAGCGCTTAAC	
dfrA50-	AAGAAGGAGATATACATATGAAGATG	To amplify the *dfrA50*

pET21b-F	ATCGCCGCAGTTG	gene for ligation into pET21b(+)
dfrA50-pET21b-R	TGGTGATGGTGATGCTCGAGACTGCGACGATAAACAATCT	
dfrA51-pET21b-F	AAGAAGGAGATATACATATGAAGATGATCGCAGCAGTGGG	To amplify the *dfrA51* gene for ligation into pET21b(+)
dfrA51-pET21b-R	TGGTGATGGTGATGCTCGAGACTGCGACGATAAACAATCT	
T7	TAATACGACTCACTATAGGG	To verify whether the target gene is cloned into pET21b(+) by colony PCR
T7 terminator	TGCTAGTTATTGCTCAGCGG	
pACYC184-F	AAGAGATTACGCGCAGAC	To verify whether the target gene is cloned into pACYC184 by colony PCR
pACYC184-R	GTGATGTCGGCGATATAGG	
dfrA50-A6V-F	GATGATCGCC**GTT**GTTGGCCGTAATTATGAA	The primers for introducing the A6V mutation in *dfrA50*. The mutation changes alanine (A) to valine (V) at position 6 of DfrA50.
*dfrA50*_A6V-R	TACGGCCAAC**AAC**GGCGATCATCTTCATATG	
*dfrA50*_I13A-F	TAATTATGAA**GCC**GGTCGTGGTAATGAACTG	The primers for introducing the I13A mutation in *dfrA50*. The mutation changes isoleucine (I) to alanine (A) at position 13 of DfrA50.
*dfrA50*_I13A-R	TACCACGACC**GGC**TTCATAATTACGGCCAAC	
dfrA50-E26A-F	CTGCCCGAGC**GCC**CTGAAACTGTTTCGTGAAC	The primers for introducing the E26A mutation in *dfrA50*. The mutation changes glutamic acid (E) to alanine (A) at position 26 of DfrA50.
dfrA50-E26A-R	ACAGTTTCAG**GGC**GCTCGGGCAGCGCCACGG	
*dfrA50*_S48A-F	AACCATGGAA**GCC**CTGAAACGTCCGCTGCCG	The primers for introducing the S48A mutation in *dfrA50*. The mutation changes serine (S) to alanine (A) at position 48 of DfrA50.
*dfrA50*_S48A-R	GACGTTTCAG**GGC**TTCCATGGTTTTACGACC	
dfrA51-A6V-F	GATGATCGCA**GTGGTG**GGCCGTAATTATGAA	The primers for introducing the A6V mutation in *dfrA51*. The mutation changes alanine (A) to valine (V) at position 6 of DfrA51.
*dfrA51*_A6V-R	TACGGCCCAC**CAC**TGCGATCATCTTCATATG	

dfrA51-I13A-R	TACCAATACC**GGC**TTCATAATTACGGCCCAC	The primers for introducing the I13A mutation in *dfrA51*. The mutation changes isoleucine (I) to alanine (A) at position 13 of DfrA51.
dfrA51-I13A_F	TAATTATGAA**GCC**GGTATTGGTAACGAGCTG	
dfrA51-D26A-F	CTGCCCTACC**GCC**CTGAAGTTATTCAAACAG	The primers for introducing the D26A mutation in *dfrA51*. The mutation changes aspartic acid (D) to alanine (A) at position 26 of DfrA51.
dfrA51-D26A-R	ATAACTTCAG**GGC**GGTAGGGCAGCGCCACGG	
dfrA51-S48A-F	AACCATGGAA**GCC**CTGAAACGTCCGCTGCCG	The primers for introducing the S48A mutation in *dfrA51*. The mutation changes serine (S) to alanine (A) at position 48 of DfrA51.
dfrA51-S48A-R	GACGTTTCAG**GGC**TTCCATGGTTTTGCGGCC	

The underlined section of each primer denotes the restriction site or the homologous sequence on the plasmid used for recombination. The mutated bases highlighted in red bold in the primers represent the mutated bases corresponding to the mutated amino acids in the protein sequences.
